# Effects of* Streptococcus bovis* Isolated from Bovine Rumen on the Fermentation Characteristics and Nutritive Value of Tanzania Grass Silage

**DOI:** 10.1155/2016/8517698

**Published:** 2016-03-17

**Authors:** Anderson de Moura Zanine, Emerson Alencar Bonelli, Alexandre Lima de Souza, Daniele de Jesus Ferreira, Edson Mauro Santos, Marinaldo Divino Ribeiro, Luiz Juliano Valério Geron, Ricardo Martins Araujo Pinho

**Affiliations:** ^1^Department of Animal Science, Federal University of Maranhão, 65500-000 Chapadinha, MA, Brazil; ^2^Department of Animal Science, Federal University of Mato Grosso, 78735-901 Rondonópolis, MT, Brazil; ^3^Department of Animal Science, Federal University of Paraíba, 58051-900 Areia, PB, Brazil; ^4^Department of Animal Science, Federal University of Goiás, 74690-900 Goiânia, GO, Brazil; ^5^Department of Animal Science, State University of Mato Grosso, 78250-000 Pontes e Lacerda, MT, Brazil

## Abstract

This study aimed to evaluate the effects of* Streptococcus bovis* on the fermentation characteristics and nutritive value of Tanzania grass silage. Tanzania grass was chopped and left untreated (U) or treated with* Streptococcus bovis* JB1 at 1 × 10^6^ colony-forming units per gram (cfu/g) of fresh forage or* Streptococcus bovis* HC5 at 1 × 10^6^ cfu/g of fresh forage and packed into sixtuplicate laboratory silos. The largest number of enterobacteria, molds and yeast (M&Y) occurred in untreated silages and the smallest populations of enterobacteria and M&Y and the largest numbers of lactic acid bacteria (LAB), at 9.81 and 9.87 log cfu/g, were observed in* Streptococcus bovis* JB1 and HC5, respectively (*P* < 0.05). Silages treated with JB1 and HC5 had lower (*P* < 0.05) silage pHs and concentrations of ammoniacal nitrogen (NH_3_-N) than untreated silages. The application of* Streptococcus bovis* JB1 and HC5 resulted in fewer losses through gases and effluents (*P* < 0.05), which resulted in greater dry matter recovery (DMR) and crude protein recovery (CPR) (*P* < 0.05).* Streptococcus bovis* JB1 and HC5 improved the fermentative profile and increased the concentration of crude protein and DMR and CPR in Tanzania grass silage.

## 1. Introduction

Among the tropical grasses, Tanzania grass (*Panicum maximum*) has high productive potential and a good chemical composition. However, when ensiled at an early stage of plant development, it has a high nutritional value but low DM content (less than 200 g kg^−1^), raising the buffer power, and a low concentration of water-soluble carbohydrate (less than 50 g kg^−1^ DM), which makes it difficult to obtain good-quality silage [1, 2]. When compacted in silage, plants with a low DM content produce greater quantities of effluent, which removes a large percentage of its nutrients.

Inoculations of live* Lactobacillus *sp.,* Pediococcus *sp., and* Streptococcus *sp. cultures that convert sugars (fructose or glucose) into lactic acid decrease fermentation losses [3]. Consequently,* Streptococcus bovis*, a LAB isolated from the rumen, may be a viable alternative for the production of perennial grass silage.

The main characteristic of this species is its specific growth speed, which is 30% greater than that of the aforementioned lactic acid-forming bacterial species used as silage inoculants. This suggests that it can act as a fermentation starter culture by promoting a rapid reduction in silage pH [4–6] with benefits that include an improved fermentation profile and increased nutritive value as well as a reduction in nutrient losses through gases and effluent.

The objective of this study was to evaluate the effects of* Streptococcus bovis* JB1 and HC5 on the fermentation characteristics and nutritive value of Tanzania grass silage.

## 2. Materials and Methods

The experiment was carried out at the Department of Animal Science at the Federal University of Viçosa, which is located in the municipality of Viçosa, MG, Brazil, during the summer (rainy) season. Viçosa is situated at 20° and 45′ south latitude and 42° and 51′ west longitude at an altitude of 657 m. The mean annual rainfall is 1341 mm, 86% of which falls from December 2008 to April 2009.

An established Tanzania grass (*Panicum maximum* cv. Tanzania) meadow of approximately 0.5 ha in size was used for the study. After a standardizing cut, the meadow was fertilized with nitrogen and potassium in the form of ammonia sulfate and potassium chloride, respectively, and the grass was harvested after 65 days.

Tanzania grass was harvested and chopped to a theoretical length of 2 cm with a forage harvester, and, within 30 min of harvesting, the chopped grass was divided into three 30 kg piles. Each pile was assigned to one of the following treatments: deionized water, untreated (U);* Streptococcus bovis* JB1 applied at 1 × 10^6^ cfu/g of fresh forage; and* Streptococcus bovis* HC5 applied at 1 × 10^6^ cfu/g of fresh forage. All treatments were dissolved in 500 mL of deionized water and uniformly sprayed on the forage under constant mixing. Both the* Streptococcus bovis* JB1 and HC5 strains were isolated in the Laboratory of Anaerobic Microorganisms of the Microbiology Department of the Federal University of Viçosa.

To prepare the inoculant, the cultures were thawed, grown in MRS culture medium (de Man, Rogosa, and Sharpe) at 39°C and submitted to three successive activations at 24-hour intervals a few days prior to the day of ensiling. Later, they were cultured in solid MRS (ranging from 10^−1^ to 10^−9^) at the same temperature for 48 h to count the* Streptococcus bovis* populations. The dilution necessary for each inoculate to reach 10^6^ cfu/g fresh forage was determined based on the bacterial concentration of each inoculant from the MRS agar culture medium. Before ensiling, the cultures were again activated following the same procedure and then diluted in distilled water at ensiling to reach the preestablished concentrations.

### 2.1. Microbial Populations and Fermentation Profile

The number of microbial groups in the plants and silage were counted by collecting 25 g of a compound silage sample from the six silos under each treatment, adding 225 mL phosphate buffer solution and blending for 1 min. Silage pH was determined immediately, and a portion of the silage was filtered through Whatman 54 filter paper, acidified with 50% H_2_SO_4_ to reduce the pH of the extract, and frozen before analysis for NH_3_-N [7].

The microbial populations of the forages and silages were also analyzed; LAB were enumerated in triplicate by pour plating using de Man, Rogosa, and Sharpe MRS agar. Agar plates were incubated anaerobically for 48 h at 39°C, and molds and yeasts (M&Y) were enumerated in triplicate by pour plating with potato dextrose agar and aerobically incubated for 7 days at room temperature. Enterobacteria were determined on violet-red bile and anaerobically incubated for 24 h at 35°C. Plates from the appropriate dilutions were counted when they contained a minimum of 30 and a maximum of 300 colonies.

In addition, the water extracted from the silage samples was analyzed for volatile fatty acid (VFA) and lactic acid with a high performance liquid chromatographer (HPLC) (Shimadzu SPD-10) at a wavelength of 210 nm. A reverse-phase C-18 column was used with 168 kg of pressure and a flow of 1.5 mL/minute.

### 2.2. Chemical Analysis

To assess their chemical composition, fresh matter samples were analyzed for DM by drying at 105°C for 12 hours in a forced-air oven and for nitrogen concentration according to method of the AOAC (1990). The samples were also analyzed for neutral detergent fiber (NDF) and acid detergent fiber (ADF); the NDF concentration of the samples was analyzed using sulfite and amylase according to Van Soest et al. [8] and ADF as described by Robertson and Van Soest [9].* In vitro* dry matter digestibility (IVDMD) was determined following the methods of [10] by incubation in a thermostatically controlled water-circulating bath.

### 2.3. Gas and Effluent Losses

Dry matter losses from the silage via the gas and effluents were determined based on the differences between weights according to Jobim et al. [11].

### 2.4. Dry Matter and Crude Protein Recovery

The dry matter recovery was estimated as the percentage of dry matter remaining in the silo upon opening when compared with the initial dry matter. Crude protein recovery was determined similarly.

### 2.5. Statistical Analysis

All microbial data were log_10_ transformed and are presented on a wet weight basis, and chemical data are presented on a DM basis. Statistical analyses were performed using the GLM procedure of [12] for a completely randomized design. Data were analyzed using the model

(1) $$\begin{array}{c} Y_{ij}=\mu +T_{i}+e_{ij}, \end{array}$$

where *Y*_*ij*_ is the observed value; *μ* is the general average; *T*_*i*_ is the treatment (inoculant) effect; and *e*_*j*_ is the residual (error). Treatments means were compared using Tukey's test and *P* < 0.05.

## 3. Results and Discussion

The number of LAB in Tanzania grass recorded in the present study can be considered low, but it is consistent with the value found in grass silage reported by Weatherfourn [7], Pahlow [13], and Ferreira et al. [14] (Table 1). Tropical grasses have a small number of LAB, less than 6 log cfu/g fresh forage, as observed in this work, and the predominance of enterobacteria probably resulted from the low soluble sugar content and high water potential of the silage, as reported by Zanine et al. [2] and Santos et al. [15]. The use of inoculants can improve lactic fermentation by increasing the number of LAB, which inhibit the growth of enterobacteria.

A significantly larger number of enterobacteria and M&Y populations were observed in untreated silage (*P* < 0.05; Table 2) and silage inoculated with* Streptococcus bovis* HC5 and JB1 produced a larger number of LAB, 9.28 and 9.24 (log cfu/g), respectively. These results explain the lower pH and higher lactic acid concentration in the inoculated silage, as shown in Table 3.

Lower pH values were observed in silage inoculated with* S. bovis* JB1 and HC5 (*P* < 0.05; Table 3), and there was greater lactic acid production (*P* < 0.05) in the inoculated silage, 65.79 and 64.67 (g kg^−1^), respectively. These results are consistent with Muck [16], who found that greater LAB growth resulted in higher lactic acid production and that the reduction in pH reflected rapid lactic acid fermentation. The pH of a foodstuff is one of the main factors that determines the growth and survival of the microorganisms within it and is also used as a quality parameter in the ensiling process.

The chemical composition and IVDMD values of Tanzania grass (Table 4) were similar to those reported by Ferreira et al. [6], Zanine et al. [17], and Pompeu et al. [18]. Tanzania grass exhibited a low DM content at ensiling, a characteristic of warm-season grasses when they are managed for biomass quantity and quality. However, this characteristic makes it difficult to obtain good-quality silage due to the risk of secondary fermentation that occurs if grasses are ensiled immediately after cutting and without the use of additives capable of reversing the process.

The highest concentrations of crude protein were observed in silage inoculated with* Streptococcus bovis* HC5 and JB1 (*P* < 0.05; Table 5). The high crude protein concentration in treated silage can be explained by reduced proteolysis as enterobacterial growth was inhibited.

The IVDMD did not differ between treatments (*P* > 0.05), with values ranging from 618.6 to 602.4 g kg^−1^, and Zanine et al. [17] and Penteado et al. [19] also did not observe changes in the IVDMD of treated silage. Improved fermentation probably affects the chemical composition and not the digestibility of silage. According to Cezário et al. [20], the effects of inoculants on silage digestibility are still unknown, and, in the case of tropical grasses, the main benefits of inoculants are reduced losses and improved fermentation.

Applying the* Streptococcus bovis* JB1 and HC5 inoculants resulted in fewer losses through gases and effluents (*P* < 0.05; Table 6), which resulted in greater DMR and CPR (Table 6). The* Streptococcus bovis* inoculants (JB1 and HC5) were responsible for the lower gas losses, so they were the most efficient treatments for increasing DMR and CPR.

The presence of enterobacteria and M&Y is undesirable because they consume nutrients that would otherwise be available to the LAB, and they also produce toxins and a large quantity of ammonia during ensiling [20]. As shown in Table 1, they are present in forage in significant quantities before the ensiling process.

The action of* Streptococcus bovis* in reducing the pH may have supported LAB development, as their populations were larger in treated silage, and this reduction in pH is partly responsible for the decrease in the enterobacteria population (Table 2). The pH reduction in tropical grass silage inoculated with* Streptococcus bovis* JB1 and HC5 strains was reported by Ferreira et al. [14] and Oliveira et al. [21].

The action of* Streptococcus bovis* supported lactic acid production, which is a main factor in the reduction of silage pH [16]. A similar result was reported by Penteado et al. [19], who worked with Mombasa grass (*Panicum maximum *Jacq) and observed that inoculation with* Lactobacillus plantarum* to produce lactic acid improved the silage fermentation profile.

It is important to emphasize that the pH value, together with the speed at which it declines during the fermentation process, can determine the types of microorganisms that can grow and dominate the environment. Based on the results of this study and only considering pH as a factor restricting microbial growth, it can be inferred that only the untreated silage did not result in fermentation of the same quality. The pH values for obtaining good-quality silage are between 3.8 and 4.2 [3], and high pH indicates nutrient losses, mainly of proteins, that result in less palatable materials with an unpleasant smell.

One of the main alterations in silage is the increase in NH_3_-N compared to the total nitrogen. It is affected by the action of microorganisms, and this parameter, together with the concentration of organic acids and pH, is used to define the quality of the fermentation process. Regarding the concentration of NH_3_-N, the lowest values were observed in silage treated with* Streptococcus bovis* JB1 and HC5, and this reduction may have occurred due to ammonia intake, a characteristic of* Streptococcus bovis* as reported by Cezário et al. [4] and Mantovani et al. [5], or the inhibition of the proteolytic organisms due to pH reduction [16]. According to Leandro [22], a greater concentration of NH_3_-N indicates more intense proteolysis, mainly because the amino acids are fermented by the proteolytic clostridia through valine and leucine deamination and the redox reaction between alanine and glycine. For this silage to be considered of being satisfactory, acceptable, or low quality, the concentration of NH_3_-N should be lower than 10%, between 10 and 15%, and over 20% of total N, respectively [3]. Muck and Kung Jr. [23], in a review of studies involving microbial inoculants published between 1990 and 1995, emphasized that the inoculants were relatively successful in 60% of the studies and resulted in lower pH, ammoniacal nitrogen, and lactic acid predominance and, therefore, a better silage fermentation profile.

Inoculation of Tanzania grass silage increased the concentration of lactic acid and decreased the concentrations of acetic, butyric, and propionic acid (Table 3). This was consistent with [16], who observed that the addition of homofermentative lactic acid bacteria increased lactic acid production during fermentation. According to Santos et al. [15], greater lactic acid production can lead to lower dry matter losses from silage because lactic acid fermentation results in minimum losses while acetic and butyric fermentation is associated with secondary fermentation and dry matter losses in the form of gases.

The lowest concentrations of acetic, butyric, and propionic acids were also observed in the above-mentioned silage (*P* < 0.05), and the low production of acetic acid in silage might be explained by low microbiological activity of the heterofermentative bacteria throughout the fermentation process (Table 3). McDonald et al. [3] reported that high acetic acid production indicated the occurrence of enterobacterial action during the initial stages of silage fermentation and competition with LAB for nutrients.

Neumann et al. [24] found that undesirable fermentation modified the composition of silage, resulting in low intake because products such as NH_3_-N and VFA were formed with acetic acid especially negatively affecting the acceptability of silage by animals due to reduced palatability. The acetic acid-producing bacteria act first due to the presence of oxygen but are soon inhibited by the increase in the temperature and acidity of the medium.

Another disadvantage of undesirable fermentation is related to the development of Clostridia, which produce butyric acid and reduce silage conservation. Therefore, there are heavy energy losses during this fermentation process (more than 20%) compared to lactic acid fermentation, in which energy losses are reduced to less than 5% [3, 25].

The DM content of silage ranged from 182.3 g kg^−1^ (untreated silage) to 195.6 g kg^−1^ (silage treated with* Streptococcus bovis* JB1). Despite the low DM content, inoculation with* Streptococcus bovis* produced a good silage fermentation profile and increased DMR and CPR (Table 6).

Similar results were observed by Oliveira et al. [21], who reported that inoculation with* Streptococcus bovis* strains HC5 and JB1 resulted in greater silage DM content with values of 202.8, 214.2, and 214.6 g kg^−1^ for untreated silage and silage treated with* Streptococcus bovis* HC5 and JB1, respectively. Ferreira et al. [6] observed significantly greater DM content in silage treated with* Streptococcus bovis* strains HC5 and JB1 compared to the untreated silage with values of 188.8, 187.8, and 176.6 g kg^−1^, respectively (*P* < 0.05).

Together, the silage treated with both* Streptococcus bovis* JB1 and HC5 exhibited a small increase (*P* < 0.05) in the concentration of CP compared to the untreated silage. The* Streptococcus bovis* species can release a growth inhibitor of proteolytic bacteria, such as enterobacteria or Clostridia, known as bacteriocin, which decreases the losses of protein nitrogen from treated silage [5]. The greater concentration of CP in silage inoculated with* Streptococcus bovis* strains HC5 and JB1 can also be explained by the common ability of all* Streptococcus bovis* strains to synthesize protein from ammonia [21]. Untreated silage, even with a lower CP value (100.1 g kg^−1^ DM) compared to the treated silage, can be of good nutritional value considering that tropical grasses do not greatly exceed this value in the vegetative stage.

The concentrations of the fibrous fraction constituents and the IVDMD (Table 5) did not differ among treatments (*P* > 0.05).

Applying* Streptococcus bovis* JB1 and HC5 resulted in decreased gas and effluent losses and increased DMR and CPR (*P* < 0.05; Table 6). When forage plants with high moisture content are ensiled, dry matter losses by effluent production can exceed 10 kg/t, but when the dry matter content is approximately 300 g kg^−1^, effluent production is not very significant. According to Reich and Kung [26] and Daniel et al. [27], the loss of oxygen availability to the ensiled material contributed to the rupture of the plant cell membrane, facilitating water losses from the cell and effluent production in the first stages of ensiling.

The high DMR and CPR in treated silage indicated that enterobacteria, heterofermentative bacteria, and proteolytic bacteria were inhibited, so the increased losses were probably a consequence of the reduced silage pH and lower NH_3_-N production (Table 3). This likely occurred due to the greater production of lactic acid (Table 2) in silage treated with* Streptococcus bovis*.

## 4. Conclusions

Both* Streptococcus bovis* JB1 and HC5 improved the fermentative profile, increased the crude protein and dry matter contents, and improved the crude protein recovery in Tanzania grass silage.

## Figures and Tables

**Table 1:** Number of lactic acid bacteria (LAB), enterobacteria (ENT), and molds and yeasts (M&Y) in Tanzania grass before ensiling (log cfu g^−1^).

Treatments	LAB	ENT	M&Y
Untreated	4.51	4.63	5.12
JB1	4.53	4.59	5.09
HC5	4.49	4.74	5.08

Untreated: Tanzania grass (TG); JB1: TG plus *S. bovis* JB1; HC5: TG plus *S. bovis* HC5.

**Table 2:** Number of lactic acid bacteria (LAB), enterobacteria (ENT), and molds and yeasts (M&Y) in Tanzania grass silage (log cfu g^−1^).

Treatments	LAB	ENT	M&Y
Untreated	7.06^b^	3.93^a^	4.87^a^
JB1	9.24^a^	2.73^b^	2.61^b^
HC5	9.28^a^	2.81^b^	2.73^b^

VC (%)	3.25	3.22	3.19

Means within a column with different superscripts differ significantly (*P* < 0.05).

Untreated: Tanzania grass (TG); JB1: TG plus *S. bovis* JB1; HC5: TG plus *S. bovis* HC5.

**Table 3:** Mean values of pH, NH_3_-N, and lactic, acetic, butyric, and propionic acids in Tanzania grass silage.

Treatment	pH	NH_3_-N (mg/dL)	Lactic acid (g kg^−1^ DM)	Acetic acid (g kg^−1^ DM)	Butyric acid (g kg^−1^ DM)	Propionic acid (g kg^−1^ DM)
Untreated	4.60^a^	11.59^a^	50.98^b^	5.97^a^	0.39^a^	3.97^a^
JB1	4.10^b^	10.75^b^	65.79^a^	4.25^b^	0.20^c^	2.45^b^
HC5	4.23^c^	10.80^b^	64.67^a^	4.40^b^	0.22^b^	2.40^b^

VC (%)	2.03	2.39	7.17	10.22	8.01	9.69

Means within a column with different superscripts differ significantly (*P* < 0.05).

Untreated: Tanzania grass (TG); JB1: TG plus *S. bovis* JB1; HC5: TG plus *S. bovis* HC5.

**Table 4:** Chemical composition and *in vitro* digestibility of Tanzania grass before ensiling.

Treatment	DM g kg^−1^	CP g kg^−1^ DM	NDF g kg^−1^ DM	ADF g kg^−1^ DM	HEM g kg^−1^ DM	IVDMD g kg^−1^ DM
Tanzania grass	205	913.9	861	120.1	730.1	404.5

DM: dry matter; OM: organic matter; CP: crude protein; NDF: neutral detergent fiber; ADF: acid detergent fiber; HEM: hemicelluloses; IVDMD: *in vitro* dry matter digestibility.

**Table 5:** Chemical composition and *in vitro* digestibility of Tanzania grass silage.

Treatment	DM g kg^−1^	CP g kg^−1^ DM	NDF g kg^−1^ DM	ADF g kg^−1^ DM	HEM g kg^−1^ DM	IVDMD g kg^−1^ DM
Untreated	182.3^b^	100.1^b^	714.3^a^	383.4^a^	330.9^a^	618.6^a^
JB1	195.6^a^	112.5^a^	709.0^a^	371.7^a^	337.3^a^	620.4^a^
HC5	192.3^a^	111.9^a^	716.6^a^	384.5^a^	332.1^a^	628.7^a^

VC (%)	3.34	5.16	7.02	7.37	8.11	7.89

Means within a column with different superscripts differ significantly (*P* < 0.05).

Untreated: Tanzania grass (TG); JB1: TG plus *S. bovis* JB1; HC5: TG plus *S. bovis* HC5.

**Table 6:** Mean values of gas losses (GL), effluent losses (EL), dry matter recovery (DMD), and crude protein recovery (CPR) of Tanzania grass silage.

Treatment	GL (g kg^−1^)	EL (kg/t)	DMR (g kg^−1^)	CPR (g kg^−1^)
Untreated	33.8^a^	38.99^a^	750.6^b^	804.7^b^
JB1	26.5^b^	34.37^b^	769.9^a^	906.8^a^
HC5	25.9^b^	27.69^c^	772.7^a^	904.1^a^

VC (%)	8.89	6.23	6.01	3.27

Means within a column with different superscripts differ significantly (*P* < 0.05).

Untreated: Tanzania grass (TG); JB1: TG plus *S. bovis* JB1; HC5: TG plus *S. bovis* HC5.
